# Optical reciprocity induced wavefront shaping for axial and lateral shifting of focus through a scattering medium

**DOI:** 10.1038/s41598-022-10378-7

**Published:** 2022-04-16

**Authors:** Abhijit Sanjeev, Vismay Trivedi, Zeev Zalevsky

**Affiliations:** Faculty of Engineering and the Institute for Nanotechnology and Advanced Materials, Bar-Ilan University, 5290002 Ramat-Gan, Israel

**Keywords:** Optics and photonics, Applied optics, Adaptive optics

## Abstract

Light propagating along a reversed path experiences the same transmission coefficient as in the forward direction, independent of the path complexity. This is called the optical reciprocity of light, which is valid for not too intense scattering media as well. Hence, by utilizing the reciprocity principle, the proposed novel technique can achieve axially and laterally tunable focus, non-invasively, through a scattering media without a priori knowledge or modeling of its scattering properties. Moreover, the uniqueness of the proposed technique lies in the fact that the illumination and detection are on the same side of the scattering media.

## Introduction

Light passing through a scattering media such as biological tissues undergo scattering which creates difficulties for an imaging system to produce quality images. Especially while using a monochromatic laser source, random interference produces speckles. Hence it is difficult to achieve optical focusing and imaging for its internal targets, which restricts in-depth imaging for medical diagnosis and treatment^1,2^. The information gets distorted within the speckle. However, the information is not lost, it just needs to be retrieved from the scattered speckle pattern. Starting from the late 1960s, many researchers have paved the way for this desirous goal of imaging through a scattering media with includes the works of Goodman et al.^3^, Leith and Upatnieks^4^, and Kogelnik and Pennington^5^.

The Optical Coherence Tomography technique utilizes the ballistic photons (photons that are not scattered) for imaging making it an important tool in many diagnostic measurements^6,7^. However, this technique suffers from the fact that it requires a lot of image processing tools, and post processing. Also it is not very effective for thick scattering medium, especially when the thickness is larger than the mean free path as the number of ballistic photons will be very few and the signal will be corrupted by non-ballistic photons as well^9–11^.

Another interesting approach is to exploit the *memory effect* of speckles. Memory effect states that there exists a correlation between a speckle pattern even when the laser illumination is tilted within a given angular range. Freund^12–14^ used the scattering media as a lens based on speckle intensity correlation. This idea was extended by Bertolotti et. al^13^ to image a fluorescent object behind the media. Phase retrieval algorithms like Fienup’s phase retrieval algorithm^15^ were utilized by Katz et. al^16^ to retrieve the object from the autocorrelation of the speckle pattern. Singh et al^17^ introduced a point source as well in the object plane. The advantage was that with simple autocorrelation of the camera intensity, the image was reconstructed hence avoiding the use of a phase retrieval algorithm. Despite the advantage of utilizing the scattered light without any wavefront correction, this approach suffers from the limited memory effect range and axial decorrelation of the speckle effect^16^. Hence this technique is inadequate for imaging through thick specimens such as biological tissues.

Apart from these optical techniques, acoustic waves can also be used in conjunction with the optical wave for deep tissue imaging because of the deeper penetration of acoustic waves. To this end, we have techniques like photoacoustic tomography^19^, acoustic-Opto tomography^20,21^and ultrasound-guided optical imaging^21^. The aforementioned techniques are non-invasive with the capability to shift the achieved focus. However, the main disadvantage lies in the fact that the resolution of the focal spot depends on the ultrasonic wavelength is orders of magnitude inferior to the optical diffraction limit.

Optical Wavefront Shaping Technique is potentially a strong candidate for focusing light through a scattering media by shaping the incident wavefront employing some feedback from the behind of the scattering media. Vellekoop et al^22^ utilized a Spatial Light Modulator (SLM) to modulate the incoming phase of the beam. A camera was placed behind the scattering medium, and this acted as feedback to guide the wavefront pattern on the SLM. Finally, through optimization, a focused spot was obtained on the camera. Later Popoff et al^23^ came up with the idea of measuring the transmission matrix of a scattering media and used it to introduce the inverse phase on the SLM to get a focused spot behind the scattering media. This method requires an initial calibration to find the complete transmission matrix. There were several works done by using SLM as a phase compensator to image through a scattering medium ^18,24–33^. Among them, optical phase conjugation is a well-known technique wherein the light distribution is replicated by reversing the propagation direction of the detected field while conjugating its wavefront. Digital optical phase conjugation (DOPC) has been well established for focusing and imaging through complex or disordered media^34–37^.

**Figure 1:**
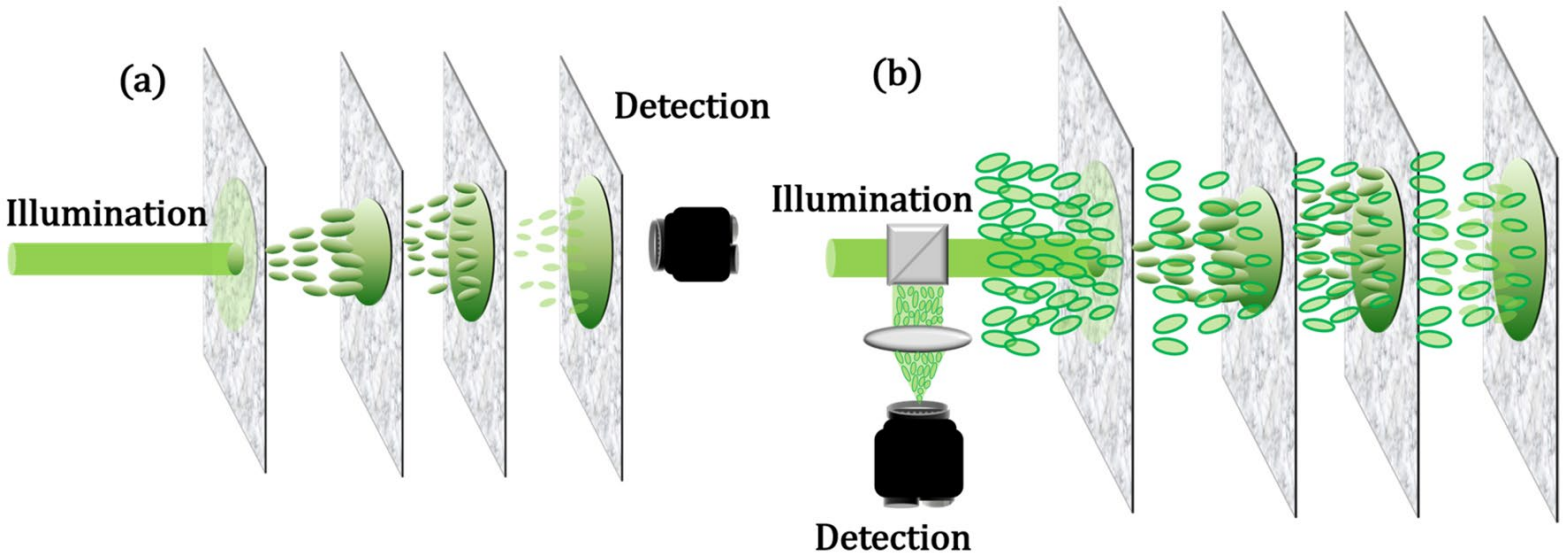
Schematic description of the proposed non-invasive technique for imaging through a scattering media (**a**) the conventional technique vs (**b**) Our technique that requires no feedback nor camera behind the media as in (**a**).

All the above-mentioned technique requires either some sort of feedback from behind the scattering media or need to know the scattering properties beforehand or need to perform some sort of post-processing algorithms (Fig. 1a). We, in our previous work^38^, laid the theoretical foundation to a novel technique of focusing light through a scattering medium non-invasively, i.e., the illumination and detection are on the same side of the media (Fig. 1b). In this paper, we extend this technique further to show that indeed it is possible to focus light non-invasively and shift the focus laterally and axially with our improved technique. It is supported by a MATLAB simulation and a proof-of-concept experiment.

## Results

As mentioned earlier, to prove the validity of our technique we show by simulation that indeed it is possible to focus and shift the same axially and laterally. In addition to that, we also performed a proof-of-concept experiment. The results of both simulation and experiments are discussed in this section.

### Simulation results

Simulations were performed in MATLAB. We followed the modeling as proposed and implemented by Zhu *et al.*^44^. In our case, a thick scattering medium of thickness *L* is modeled by a stack of *M* random uncorrelated phase plates followed by a small angular spectrum propagation. The distance between each phase plate (*dl*) such that *L* = *M.dl*. We assume that (i) the refractive index contrasts are low which makes sure that the backward scattering is negligible, (ii) diffuser thickness is smaller than the transport mean free path, (iii) the diffuser’s absorption is negligible. In our simulation, we show that (i) it is possible to focus light in a thick diffuser. For this to happen the cost function is the phase correlation between the Input wavefront and that of the conjugate of the output wavefront as described by Eq. 10 (see “Methods”). (ii)Laterally shift the obtained focus by multiplying the optimized wavefront with linear phase gradient, (iii) Axially shift the focus at a *z* plane which is *z* distance after the medium for which the cost function is described in Eq. (27) (see “Methods”).

**Figure 2:**
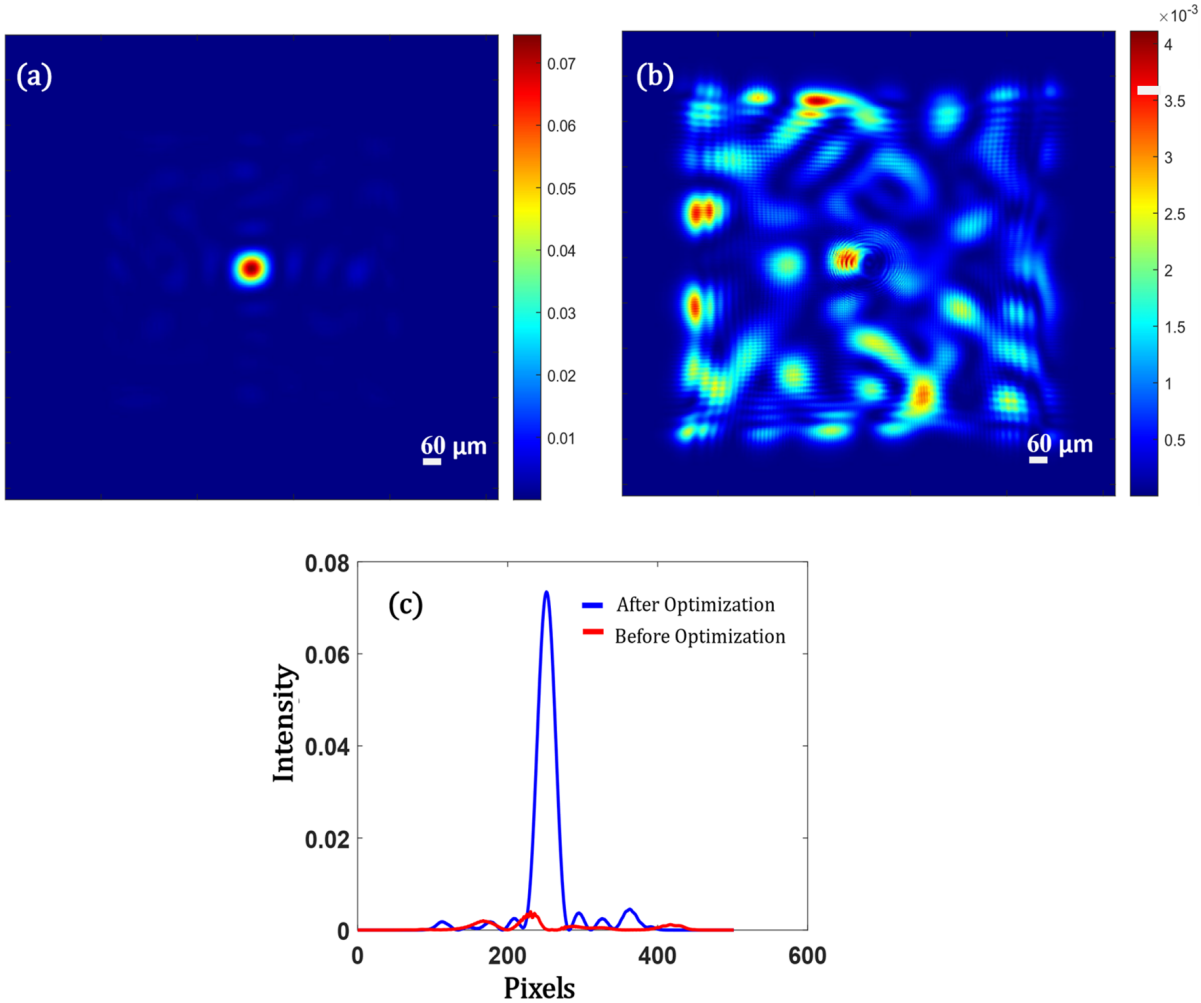
Simulation Results showing (**a**) The focus obtained after optimization when the diffuser is illuminated with the correct phase mask that cancels the diffuser to obtain a strong focus non-invasively. (**b**) Scattered Speckle pattern on the last plane of the diffuser when it is illuminated with a plane wave, (**c**) Intensity profile along an axis for both (**a**) and (**b**) marked in blue and red respectively.

Simulations were done for a total of 100 variables, being the input modes, whose values change from $$0 \;{\text{to}}\; 2\pi$$. The optimization scheme used was Particle Swarm Optimization (see “Methods”). The number of particles or the random phase mask of $$\left( {10 \times 10} \right)$$ was 20. MaxIt = 100, the medium is modeled to be 1 mm thick with 5 scattering layers with a spacing of 142.8 µm. The correlation function increases to 0.95 in 100 iterations which include 20 iterations of each swarm. Hence the total number of iterations used is 2000. Figure 2 shows the simulation result of focusing light right through the thick medium. Figures 2a,b show the speckle intensity on the plane in the first pass through the medium. The intensity profile in Fig. 2c shows that the peak to background ratio (*pbr)* has improved by $$43 $$ times. The size of the focal spot obtained is about $$61\,\upmu {\text{m}}$$. The scattering medium behaves like a lens of focal length 1 mm with a pupil size of $$10\,\upmu {\text{m}}$$ which is the effective area of illumination on the scattering medium. Hence the *N.A* of this scattering lens is 0.005, with spot size not smaller than $$\left( {\frac{\lambda f}{D} = 53\,\upmu {\text{m}}} \right)$$. Our simulation agrees with this concept as well as we got a spot of size $$61\,\upmu {\text{m}}$$.

**Figure 3:**
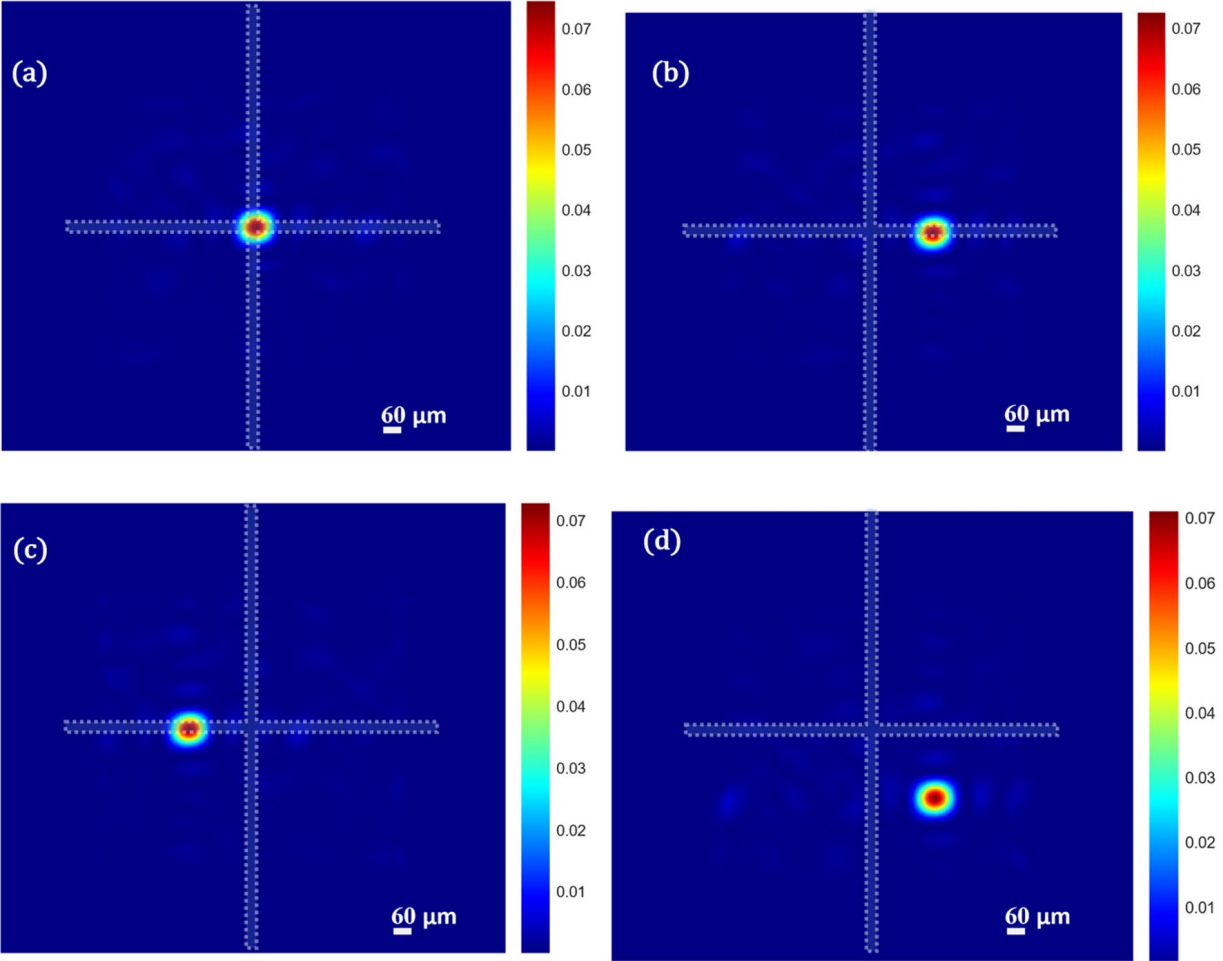
Simulation results for (**a**) focus obtained at the last diffuser plane (**b**) Shift in focus when the optimized mask is multiplied by a linear phase shift in positive x-direction, (**c**) Shift in focus when the optimized mask is multiplied by a linear phase shift in negative x-direction (**d**) Shift in focus when the optimized mask is multiplied by a linear phase shift both in x and y directions.

We proceed further in shifting the focus obtained laterally by multiplying the phase mask obtained through optimization with the required phase gradient phase mask. The results are shown in Fig. 3. To understand the shift, in each image we have added a cross wire which is centered on each image. Figure 3a shows the focus at the center, (b) lateral shift in positive x-axis f about $61\,\upmu {\text{m}}$, (c) lateral shift in negative x-axis f about $61\,\upmu {\text{m}}$, (d) combined lateral shift in x and y of about $62\,\upmu {\text{m}}$ respectively. In all the cases the *pbr* remains the same.

**Figure 4:**
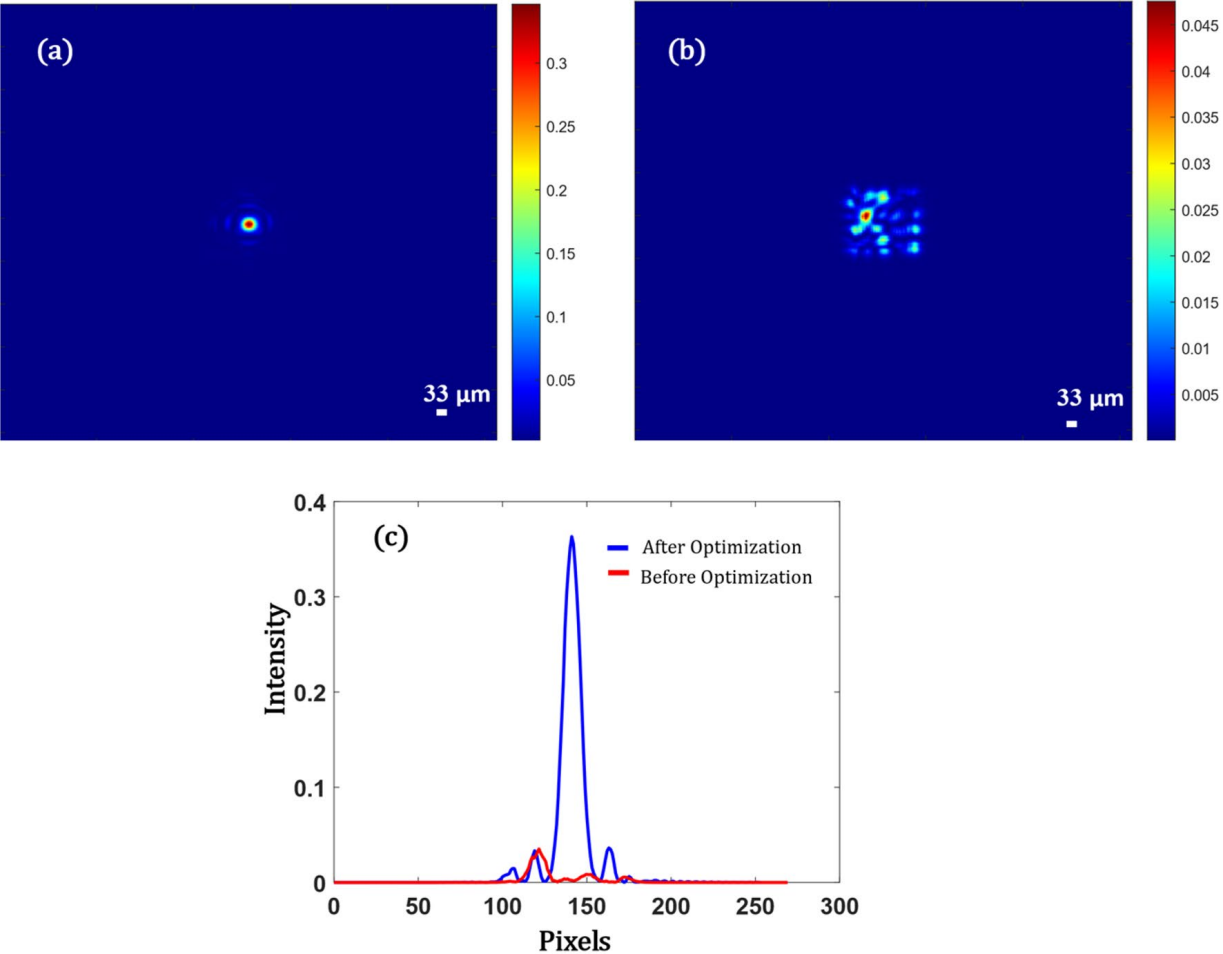
Simulation results showing, (**a**) Focus obtained on a different z plane, when the diffuser is illuminated with the optimized mask, (**b**) speckle pattern on the current z plane before optimization, (**c**) shows the profile view of the intensity pattern obtained in (**a**) and (**b**).

Axial shifting of focus is obtained at a plane $z = 0.5\,{\text{ mm}}$ from the last plane of the diffuser. This is achieved by utilizing the Eq. (27) (see “Methods”) as the cost function in the optimization. Figure 4 shows the focal spot shifting axially. The spot size is approximately $33 \,\upmu {\text{m}}$. The spot size is smaller because we tune the system such that the beam travels an axial distance $z = 0.5 \,{\text{mm}}$ from the last scattering layer and focuses light. Hence, the effective NA is higher now compared to the case where $z = 0$. In this case, the *pbr* is $30$ times higher than the scattered case.

## Experimental results

**Figure 5:**
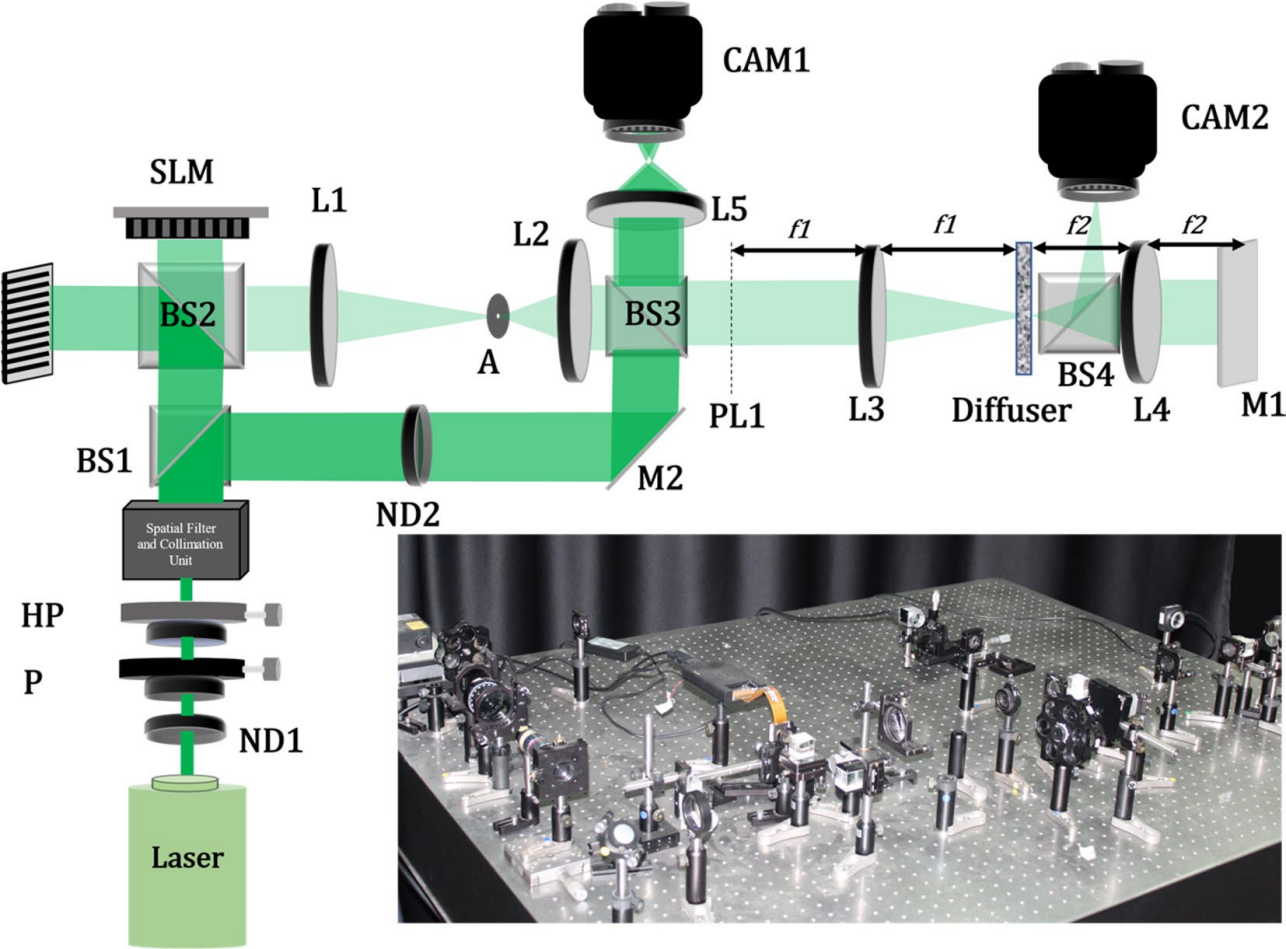
Schematic of the Experimental Setup. (Actual Pic of the Setup in the inset).

As a proof of concept and to validate our proposed novel concept, we demonstrated focusing of light through a scattering medium (Thorlabs Ground Glass Diffuser with 600 Grits). Here we also show the capability of the technique to scan the spot both axially and laterally. Figure 5 shows the schematic along with the actual experimental setup used. The experimental setup consists of a 532 nm spatially filtered collimated laser illuminating the Phase Only SLM (Holoeye-Pluto 2(1080 × 1920), pixel size 8 µm). The SLM surface is imaged to the plane PL1 using a 4f. system that consists of lens L1 (f = 200 mm), L2 (f = 100 mm), and an aperture A in the Fourier plane of lens L1 to filter out higher-order diffraction from the SLM. Further, the diffuser is placed at the focal point of the lens L3 (f = 150 mm). The lens L4 (f = 50 mm) performs the Fourier transform of the back surface of the diffuser to the Mirror M1 which reflects the light back through the same diffuser.

Lens L5 images the plane PL1 on the CAM1 (basically L5 images the output speckle reaching on the plane PL1 after the dual pass through the diffuser). The CAM1 is placed such that it is pixel to pixel matched with the SLM projection on the plane PL1. We use an off-axis reference beam to capture the interference pattern of the output speckles on CAM1 and process it using the off-axis holographic technique to retrieve the output speckle phase. This acts as feedback to the SLM. CAM2 is placed at varying z distances from the diffuser to verify whether we achieved focus at the desired location or not. However, we don’t use any information from this camera to modulate the input SLM phase.

**Figure 6:**
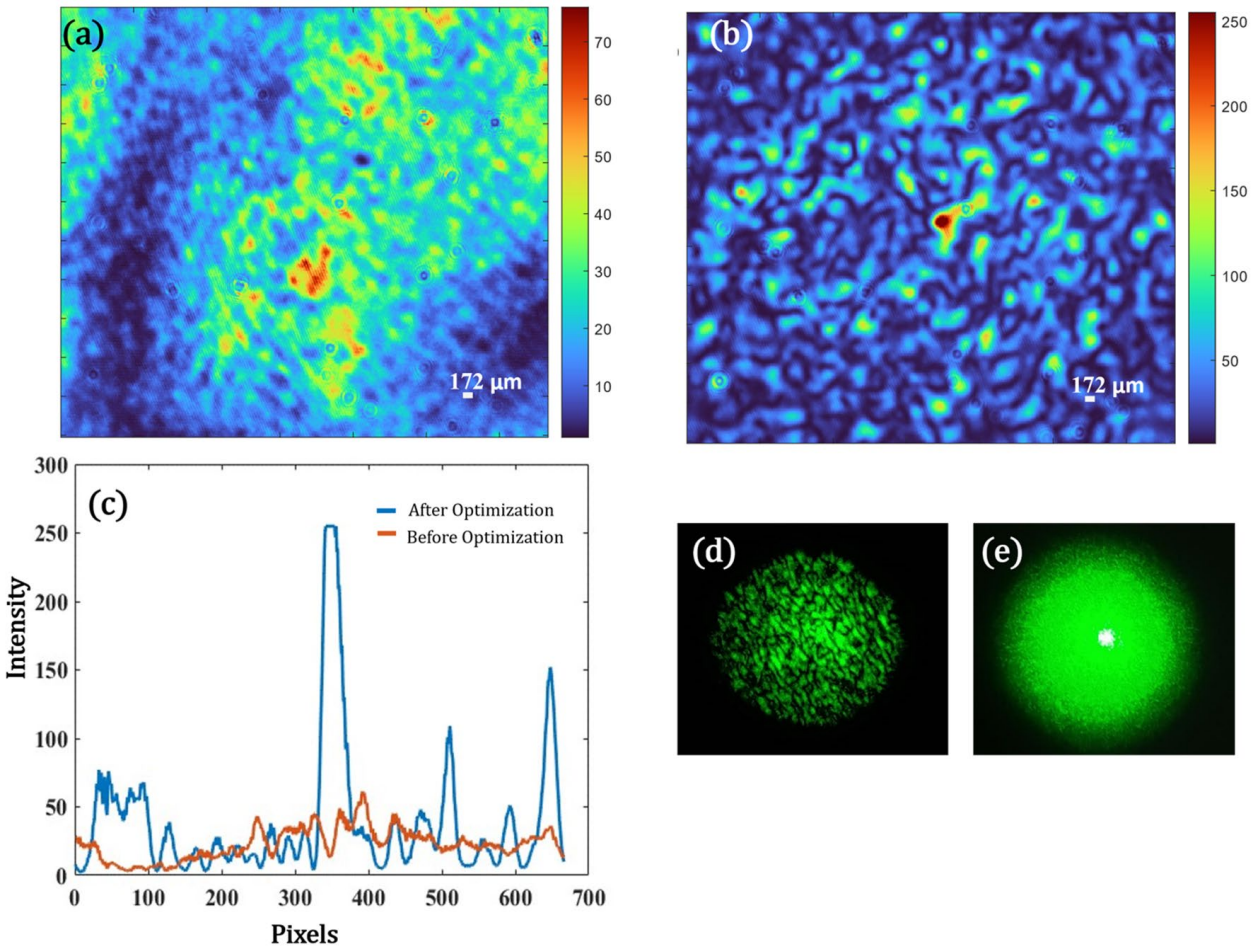
(**a**) Scattered Speckle pattern on CAM2 when the diffuser is illuminated with a plane wave at a distance of (z = 100 mm) from the diffuser, (**b**)The optimized focus after optimization, (**c**) Intensity profile along an axis for both (**a**) and (**b**) marked in red and blue respectively, (**d**) Photo taken on a smartphone of the case (**a**) on a screen, (**e**) Photo taken on a smartphone of the case (**b**) on a screen.

In the first set of experiments, we tried to focus light at a distance of 100 mm from the diffuser. The objective function to optimize the wavefront to achieve desired focus non-invasively is obtained from Eq. 27 (Please see “Methods”). The effective area illuminated on the SLM is 8.6 mm which is a square of 1080 × 1080 pixels. However, in the experiment, we bin together 27 × 27 pixels as one pixel to reduce the computational load. Hence the effective size of the input matrix becomes 40 × 40. The optimization algorithm searches for the best solution that satisfies Eq. 27. Figure 6 shows the results for this set of experiments. The speckle intensity captured on the CAM2 before the optimization is shown in Fig. 6a. Figure 6d is the image captured using a smartphone by placing a screen on the same plane as of CAM2. This shows that indeed before optimization the distribution of the speckle field is over a larger area and CAM2 captures only a portion of this field. After optimization, we achieve a strong focus with a spot size of 172 µm (FWHM) as depicted by Fig. 6b. The profile view of both Fig. 6a,b is plotted in Fig. 6c. The *pbr* has increased 6 times compared to the unfocused condition. Figure 6e shows the photo captured using a smartphone by placing a screen on the same plane after optimization. Comparing Fig. 6d,e, we see that the entire speckle energy is concentrated in the center after the optimization and there is a focal spot (Fig. 6b) within the same.

**Figure 7:**
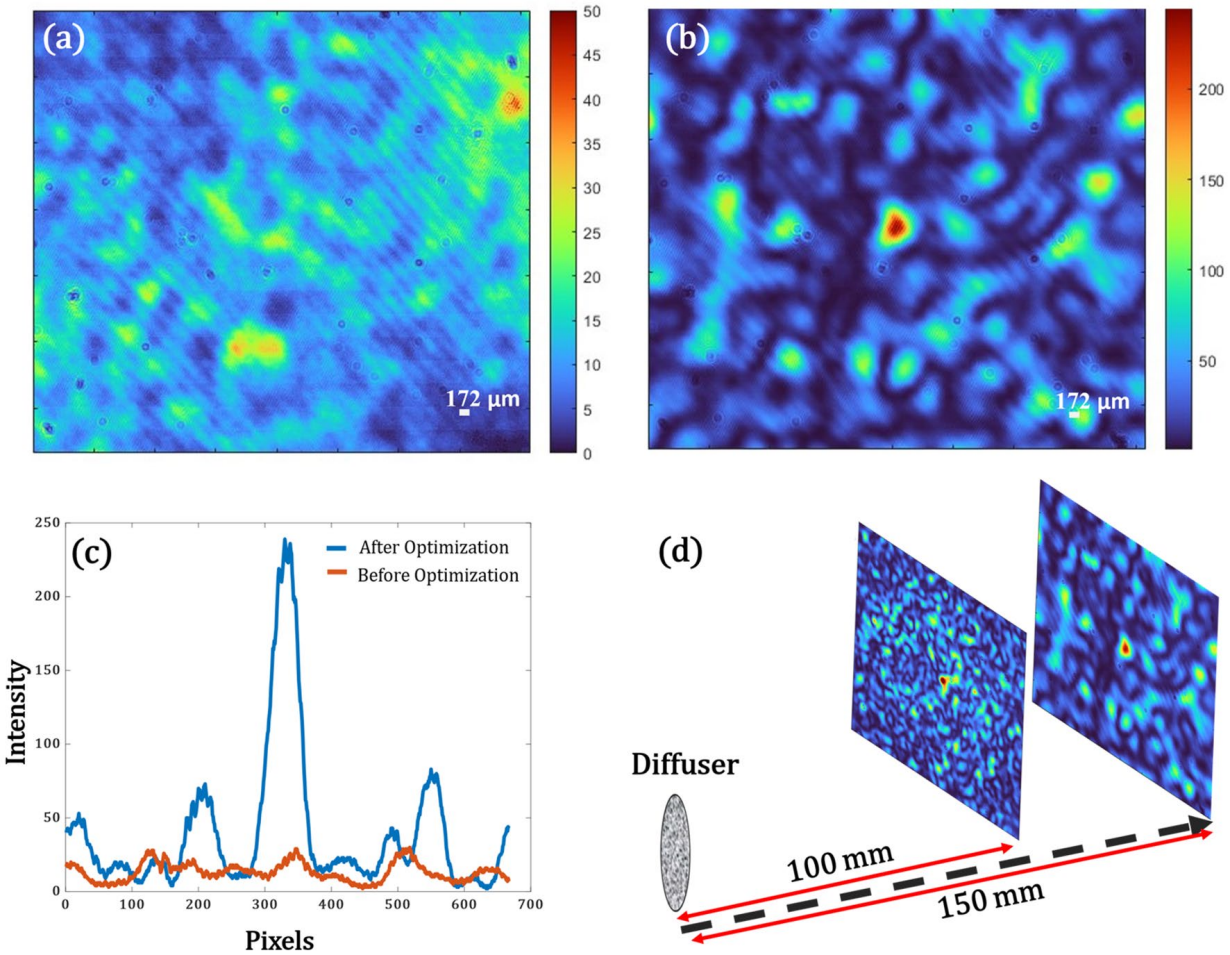
(**a**) Scattered Speckle pattern on CAM2 when the diffuser is illuminated with a plane wave at (z = 150 mm) from the diffuser, (**b**)The optimized focus after optimization, (**c**) Intensity profile along an axis for both (**a**) and (**b**) marked in red and blue respectively, (**d**) focal spot scanning along the z axis at z = 100 mm and z = 150 mm.

Second set of experiments are performed to scan the focal spot in axially. Again, using Eq. 27, changing the value of z to 150 mm, and performing an optimization based on the new condition, we were able to shift the focus to a plane at distance of 150 mm (z = 150 mm) from the diffuser. As it can be seen in Fig. 7a, the non-optimized case has scattered speckles, however, after optimization in Fig. 7b, we obtain a strong focus with a spot size of 286 µm (FWHM). Figure 7c shows the intensity profile (a) and (b). The *pbr* has improved almost 5 times the unfocused case. Here also we have effectively used 40 × 40 pixels on the SLM by binning 27 × 27 as one pixel. Figure 7 (d) depicts that the results we obtained in a simpler manner that we were able to scan the spot in z at different distances from the diffuser.

**Figure 8:**
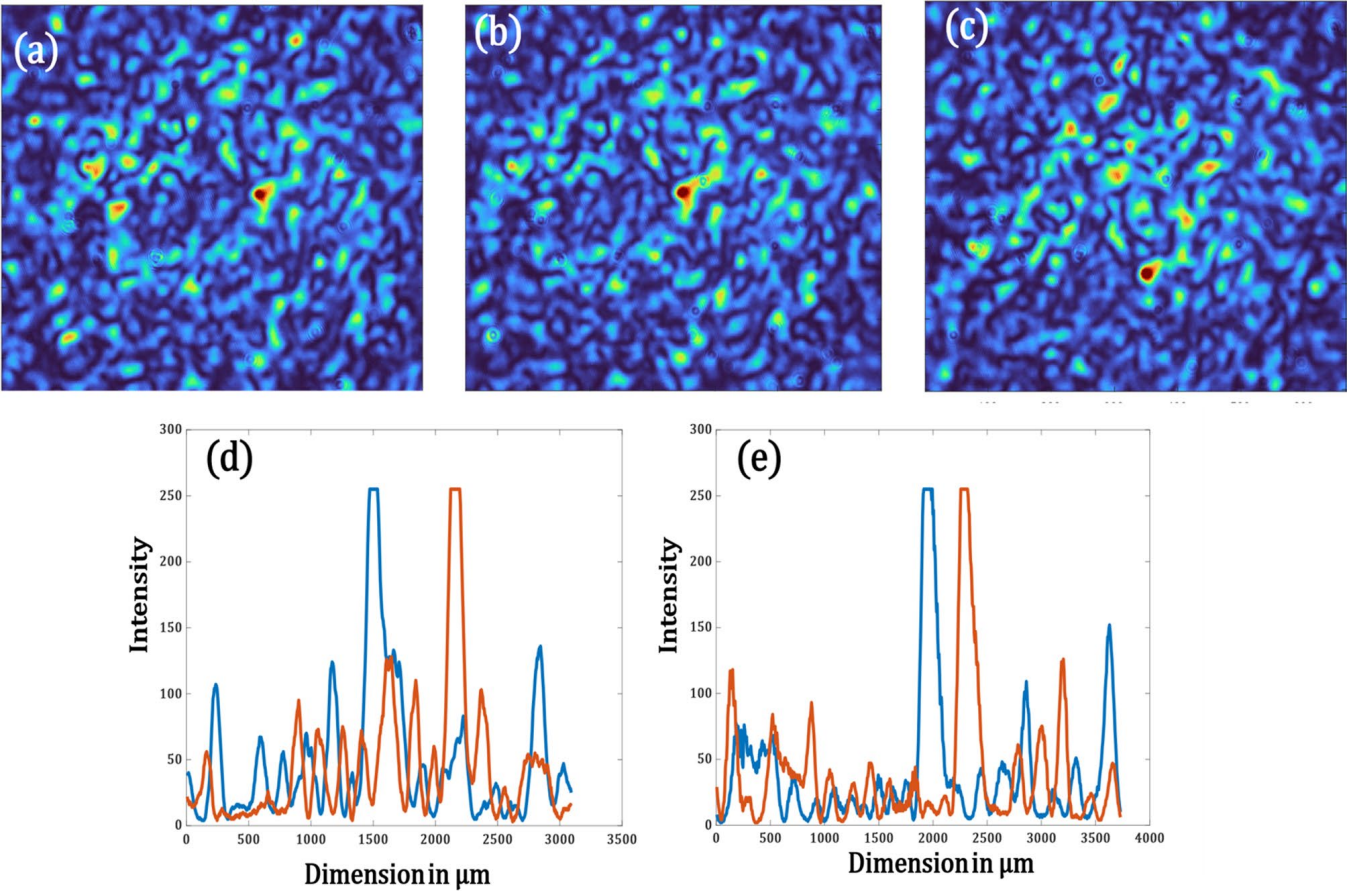
(**a**) Shifted focal spot along x, (**b**) Actual focal spot after optimization, (**c**) Shifted focal spot along y, (**d**) Profile view of (**b**) and (**a**) in blue and red respectively, the plot shows a shift of 348 µm, (**e**) Profile view of (**b**) and (**c**) in blue and red respectively, the plot shows a shift of 667 µm.

For most biological applications like imaging a specimen through a scattering tissue, it is important that the produced focal spot can shift within a desired field of view. Hence, in the next set of experiments, we show that it is possible to shift the obtained focal spots laterally as well. This can be done either by multiplying the optimized phase mask obtained with a linear grating or by shifting the pattern on the SLM which in turn will shift the focal spot due to the memory effect of speckles. We use the results from the first set of experiments we performed, i.e. by shifting the optimized phase mask used to produce the focal spot in Fig. 6b, we can shift the focal spot. We can shift to a maximum of 348 µm from the initial focus spot on both sides along the x-direction. In the y-axis, we can shift to a maximum of 667 µm in both directions from the initial focal spot. Figure 8a,c show the shift both in x and y respectively compared to the unshifted case as in Fig. 8b. The profile plots in (d) and (e) show the shifts in microns with respect to the original spot in (b).

In the above set of experiments, we concentrated on proving the concept of axial and lateral shifting of focus through a scattering medium non- invasively. We believe that there is room for improvement and future work to be done to study various parameters that need to be tuned to further improve the results. Currently, we used a thin scattering medium as a specimen. We required nearly 4000 iterations to perform the optimization. It took about 25 min for optimization since our SLM has a very slow response time (120 ms), and additionally, the phase becomes stable only after a total of 300 ms. So, for each random phase mask on the SLM, we need to wait around 300 ms for capturing the frame. The camera capturing process and the hologram reconstruction process altogether also takes time which is about 50 ms. Hence it delays the entire process by 350 ms. It was observed that the laser loses its phase stability from the initial state in about 20 min. Hence, it is essential to perform the entire optimization within this time frame. However, this is not a limitation to the technique. Today’s market is equipped with SLM that has a very fast response time of 5 to 10 ms. Also, it is important to mention the use of a Digital Micromirror Device (DMD) based system which is an array of micrometer-sized mirrors that can be individually controlled at a high refresh rate of up to 20 kHz could also be used to shape the wavefront. In addition to this, parallel processing and GPU-based programming can further boost the speed of the entire process. Indeed, using our technique, it is possible to focus light through on a thick specimen as well, but it will require more iteration and computational load as we will require more pixels on the SLM to efficiently control the phase of the light. We would like to extend our workflow towards this direction by optimizing our current setup that would accommodate the high-speed processing required for a thick specimen. We also hope that the current study encourages the research community to explore more in this domain.

## Methods

### Principle

**Figure 9:**
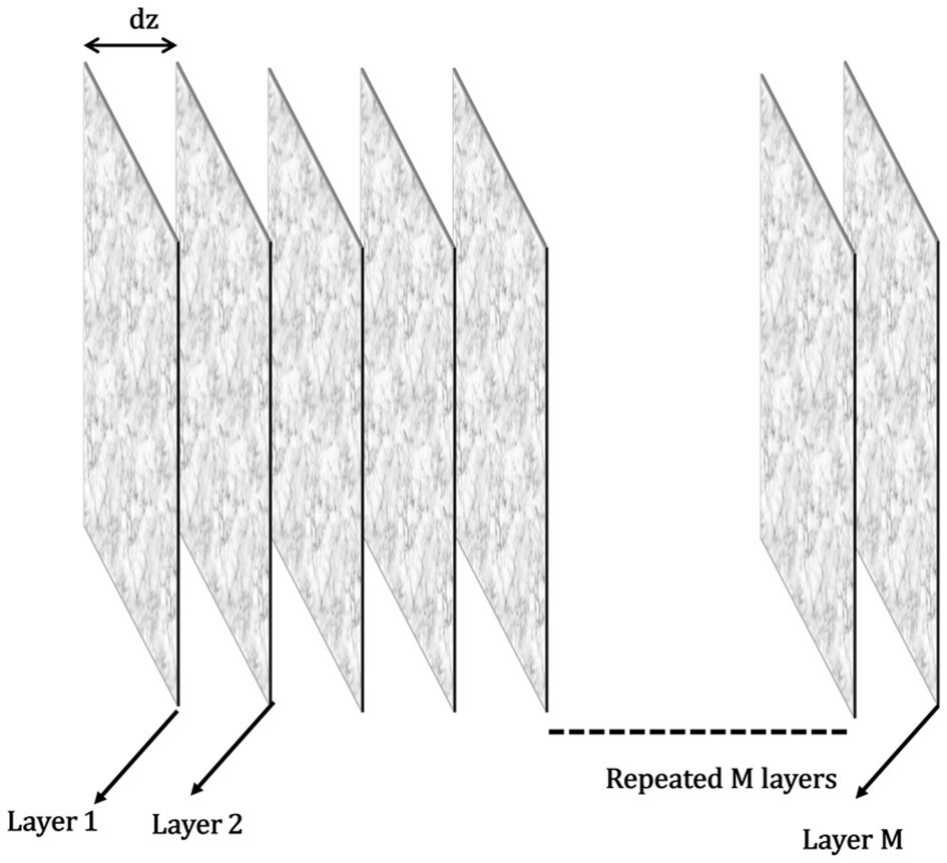
Modelling a thick scattering medium as a stack of phase plates followed by a small frees-pace propagation.

The bi-directional transmission through photonic systems is governed by the universal Lorentz reciprocity (or the Helmholtz reciprocity), which states that light propagating along a reversed path experiences the same transmission coefficient as in the forward direction, independent of the path complexity^39,40^ or the presence of loss^41–43^ In the linear regime, this suggests a definite relation, or symmetry, between the forward and the backward transmission when interchanging the source and detector. This symmetry is also valid for a forward and backward scattering of light in scattering media. Recently Szu *et al.*^8^ proved this point experimentally in a disordered medium like a multimode fiber. He measured the transmission matrix both in the forward and backward direction and found that indeed the symmetry exists, and the backward transmission matrix is the transpose of the forward transmission matrix. This lays the foundation for our technique. We employ an adaptive optics scheme-based detection for wavefront compensation to focus light in the scattering medium. In our scheme, we optimize the input wavefront applied to a scattering medium by measuring the phase of the light that passed twice through the same medium (on the way forward and on the way back). To obtain the encoded inverse wavefront that will be focused after passing the above-mentioned scattering medium we use the following modeling: we assume that the scattering medium can be modeled by a sequence of operator multiplications while the first is an operator of a random phase and the second is an operator of short free-space propagation of distance of *dz* being the average scattering length in the inspected tissue. This couple of operators is repeated until the number of repeats equals *M* = *L/dz* where *L* is the distance of the target behind the media from the point of illumination (illustration in Fig. 9).

Thus, by modeling the 1D case due to cause of simplicity, the forward scattering matrix *S* can be written as:1where F is the DFT matrix with $\left[ {F_{ij} } \right]$ and $\left[ {F_{ij} } \right]^{*}$ being Fourier and inverse Fourier matrix., λ is the optical wavelength, *z* is the free space distance, *dz* is the averaged free optical path (average distance between scattering events), P is a random phase factor (of the scatterer) and μ is the spatial frequency.

Hence,

2 $$ E_{{out_{j} }} \left( {x_{j} } \right) = S^{t} *S*E_{{in_{j} }} \left( {x_{j} } \right) $$

It is to be noted that the matrix *S* is unitary since it is the product of the multiplication of two unitary matrices. The operator matrix *S* was applied twice as there is double passing through the scattering medium (on the way forward and on the way back). On the way back, the scattering is in inverse order, and therefore the transpose operation was applied on the matrix *S*. We want to find the input field distribution vector E_in_ (which e.g., can be a phase-only distribution) such that after one passage a focus is obtained, i.e., the operator *S* produces a delta function in the middle of the spatial axis (zero position coordinate):

3 $$ \left[ {\begin{array}{*{20}c} 0 \\ 0 \\ 1 \\ 0 \\ 0 \\ \end{array} } \right] = S*E_{{in_{j} }} \left( {x_{j} } \right) $$

Thus, one can extract that:

4 $$ (S^{t} )^{ - 1} *E_{{out_{j} }} \left( {x_{j} } \right) = S*E_{{in_{j} }} \left( {x_{j} } \right) = \left[ {\begin{array}{*{20}c} 0 \\ 0 \\ 1 \\ 0 \\ 0 \\ \end{array} } \right] $$

and therefore:

5 $$ E_{{out_{j} }} \left( {x_{j} } \right) = S^{t} \left[ {\begin{array}{*{20}c} 0 \\ 0 \\ 1 \\ 0 \\ 0 \\ \end{array} } \right] = S^{t}_{{N/2_{J} }} $$

*N* is the number of spatial sampling points along the output axis.

From Eq. 3 E_in_ equals to the middle column of the matrix operator of inverse *S*:

6 $$ E_{{in_{j} }} \left( {x_{j} } \right) = S^{ - 1} *\left[ {\begin{array}{*{20}c} 0 \\ 0 \\ 1 \\ 0 \\ 0 \\ \end{array} } \right] = S^{ - 1}_{{\frac{N}{2}_{j} }} $$

Since *A* is a unitary matrix then there is a known relation between *A* transpose (*S*^t^) and inverse *S* (*S*^*-1*^).

7 $$ S^{ - 1} = S^{*} $$

where * applied on a matrix *S* denotes its conjugate transpose. By combining Eqs. 5, 6 and 7, we have

8 $$ E_{{out_{j} }}^{*} \left( {x_{j} } \right) = E_{{in_{j} }} \left( {x_{j} } \right) $$

**Figure 10:**
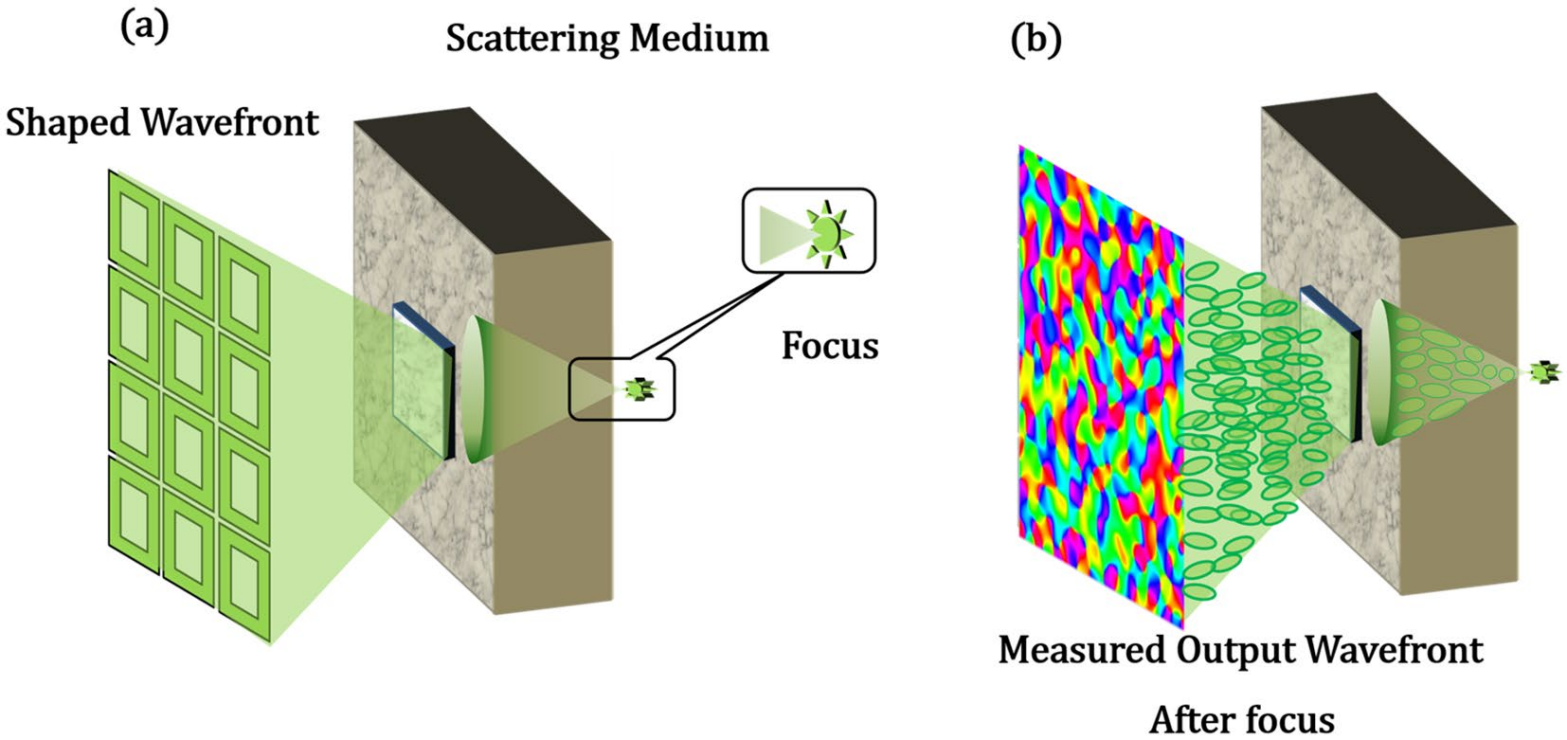
(**a**) This shows that there exists an ideal-shaped wavefront that produces a focused spot behind the thick media. (**b**) The illustration shows that the focused light passes through the same scattering medium in a backward scattering direction. The output wavefront is measured will be the conjugate of the ideal shaped wavefront. This would be the condition to obtain focus after the first pass through the media.

Hence, we obtain the desired $E_{{in_{j} }} \left( {x_{j} } \right)$ to be applied on the SLM for having a focused spot after the first passage through the medium. A pictorial representation of the above presented mathematical steps can be seen as two steps described in Fig. 10a,b.

**Figure 11:**
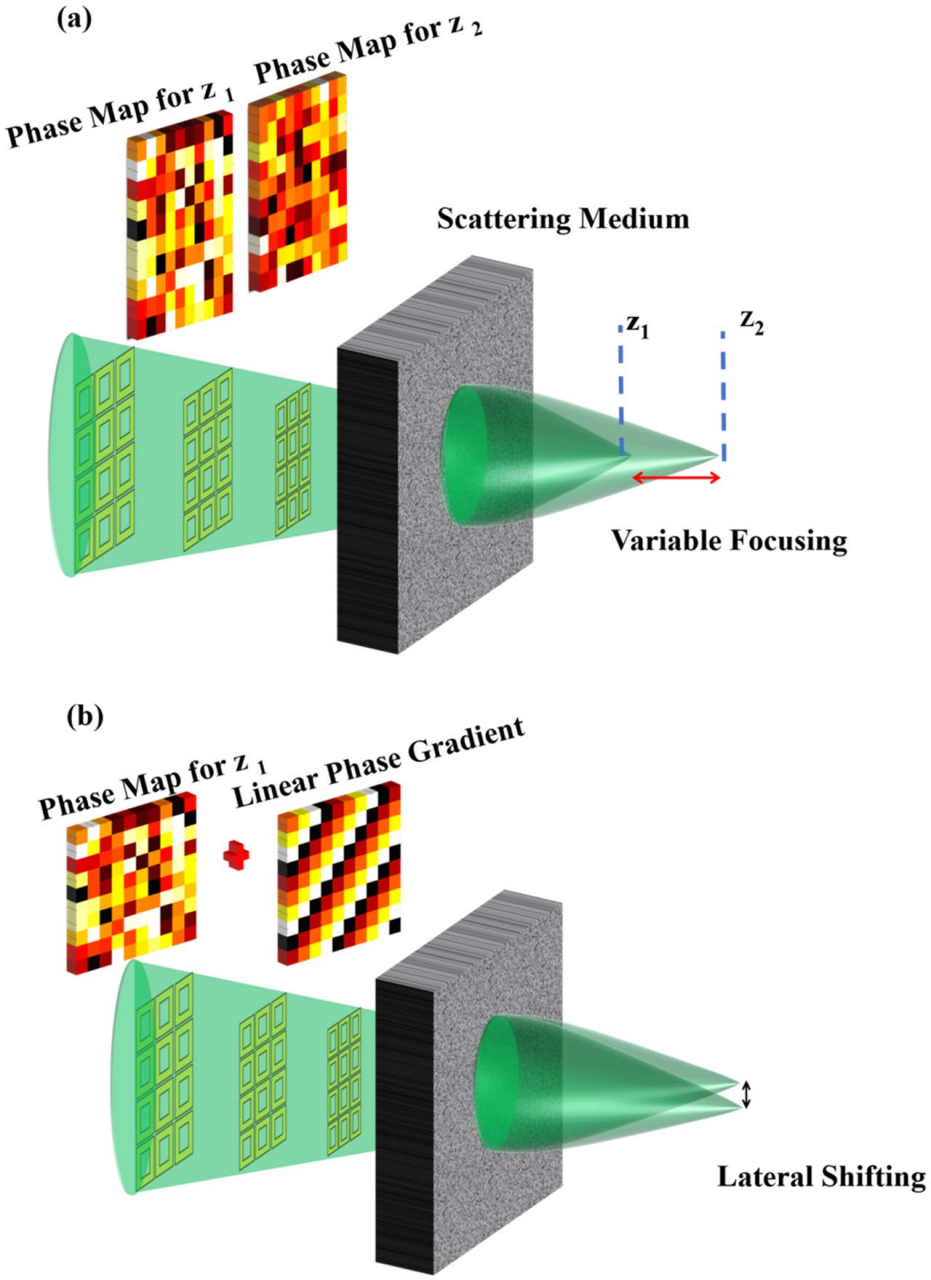
(**a**) Illustration of the Axial Tuning of focus on two different planes using the corresponding optimized phase map, (**b**) Illustration of Lateral shifting of focus by applying a linear phase gradient to the optimized mask.

We extend our technique to axially tune the focus. In the following section, we will model the theoretical foundation for axial tuning of the focal point after the diffuser. (See Illustration in Fig. 11a). We denote matrix *T* for free-space propagation of distance of $$z$$ after the scattering medium with a thickness of ($$dz M)$$. The medium has an M layer of scattering. Now the total forward scattering with the additional z propagation is given by:

9 $$ S\prime = \left[ {F_{ij} } \right]^{*} \left[ {\begin{array}{*{20}c} {e^{{ - i\pi \lambda z\mu_{j}^{2} }} } & \cdots & 0 & 0 \\ \vdots & \ddots & 0 & \vdots \\ 0 & \cdots & {e^{{ - i\pi \lambda z\mu_{j}^{2} }} } & 0 \\ 0 & 0 & 0 & {e^{{ - i\pi \lambda z\mu_{j}^{2} }} } \\ \end{array} } \right] \left[ {F_{ij} } \right]*S = T*S $$

10 $$ E_{{out_{j} }} \left( {x_{j} } \right) = S^{t} *T^{t} *T*S*E_{{in_{j} }} \left( {x_{j} } \right) $$

where $E_{{out_{j} }}$ is the electrical field of light sensed by the holographic wavefront sensor.

### Axial Focus Location Tuning

As discussed before, the assumption is that the electrical field that is back-reflected passes the same medium but in the opposite sequence. This assumption can be made because the focus we have on the object is in the center of the field of view (and thus the way back of the backscattered light is exactly symmetric to the way in but in the opposite sequence of events).

We assume focus after 1st pass and thus:

11 $$ \left[ {\begin{array}{*{20}c} 0 \\ 0 \\ 1 \\ 0 \\ 0 \\ \end{array} } \right] = T*S*E_{{in_{j} }} \left( {x_{j} } \right) $$

$$\left[ {\begin{array}{*{20}c} 0 \\ 0 \\ 1 \\ 0 \\ 0 \\ \end{array} } \right]$$ means that a focus is obtained in the center of the field of view where $E_{{in_{j} }}$ is the electrical field that is sent into the scattering medium after being shaped by the SLM.

12 $$ E_{{in_{j} }} \left( {x_{j} } \right) = S^{ - 1} *T^{ - 1} *\left[ {\begin{array}{*{20}c} 0 \\ 0 \\ 1 \\ 0 \\ 0 \\ \end{array} } \right] $$

Then, our output field (as detected by the holographic wavefront sensor) is given by

13 $$ E_{{out_{j} }} \left( {x_{j} } \right) = S^{t} *T*\left[ {\begin{array}{*{20}c} 0 \\ 0 \\ 1 \\ 0 \\ 0 \\ \end{array} } \right] $$

Hence, we can write:

14 $$ \left( T \right)^{ - 1} *(S^{t} )^{ - 1} *E_{{out_{j} }} \left( {x_{j} } \right) = T*S*E_{{in_{j} }} \left( {x_{j} } \right) = \left[ {\begin{array}{*{20}c} 0 \\ 0 \\ 1 \\ 0 \\ 0 \\ \end{array} } \right] $$

Thus,

15 $$ S*E_{{in_{j} }} \left( {x_{j} } \right) = \left( T \right)^{ - 1} *\left[ {\begin{array}{*{20}c} 0 \\ 0 \\ 1 \\ 0 \\ 0 \\ \end{array} } \right] $$

16 $$ (S^{t} )^{ - 1} *E_{{out_{j} }} \left( {x_{j} } \right) = T*\left[ {\begin{array}{*{20}c} 0 \\ 0 \\ 1 \\ 0 \\ 0 \\ \end{array} } \right] $$

Due to the unitary property of the matrix $S$, we have

17 $$ S^{ - 1} = S^{*} $$

where * is the complex conjugate as the matrix $S$ is complex.

Thus, by dividing Eq. 15 by conjugate of Eq. 16 we obtain:

18 $$ E_{{in_{j} }} \left( {x_{j} } \right)/E_{{out_{j} }}^{ \oplus } \left( {x_{j} } \right) = T^{ - 1} *\left[ {\begin{array}{*{20}c} 0 \\ 0 \\ 1 \\ 0 \\ 0 \\ \end{array} } \right]/T^{ \oplus } *\left[ {\begin{array}{*{20}c} 0 \\ 0 \\ 1 \\ 0 \\ 0 \\ \end{array} } \right] = g\left( Z \right) $$

where ⨁ is the conjugate of the respective matrices. g(z) is a known function of z. Thus, the condition for convergence is:

19 $$ E_{{in_{j} }} \left( {x_{j} } \right) = {\text{g}}\left( {\text{z}} \right){*}E_{{out_{j} }}^{ \oplus } \left( {x_{j} } \right) $$

### Calculation of ${\varvec{g}}\left( {\varvec{z}} \right)$

20 $$ g\left( z \right) = T^{ - 1} *\left[ {\begin{array}{*{20}c} 0 \\ 0 \\ 1 \\ 0 \\ 0 \\ \end{array} } \right]/T^{ \oplus } *\left[ {\begin{array}{*{20}c} 0 \\ 0 \\ 1 \\ 0 \\ 0 \\ \end{array} } \right] $$

Let us calculate

21 $$ C_{1} = T*\left[ {\begin{array}{*{20}c} 0 \\ 0 \\ 1 \\ 0 \\ 0 \\ \end{array} } \right] $$

Our free-space propagation matrix T is given by

22 $$ T = \left[ {F_{ij} } \right]^{*} \left[ {\begin{array}{*{20}c} {e^{{ - i\pi \lambda z\mu_{j}^{2} }} } & \cdots & 0 & 0 \\ \vdots & \ddots & 0 & \vdots \\ 0 & \cdots & {e^{{ - i\pi \lambda z\mu_{j}^{2} }} } & 0 \\ 0 & 0 & 0 & {e^{{ - i\pi \lambda z\mu_{j}^{2} }} } \\ \end{array} } \right] \left[ {F_{ij} } \right] $$

So, in Eq. (21), what we do is to take the DFT of a delta function which is centered at the middle of the axis which gives us a vector with all elements being ones. The coordinates of the DFT are in the frequency domain. After its operation on the delta function, we get the result in spatial domain $x:$

23 $$ F*\left[ {\begin{array}{*{20}c} 0 \\ 0 \\ 1 \\ 0 \\ 0 \\ \end{array} } \right] = \left[ {\begin{array}{*{20}c} 1 \\ 1 \\ 1 \\ 1 \\ 1 \\ \end{array} } \right] $$

In the next step, we take the $DFT^{*}$ of the Fresnel matrix which is multiplied by the vector of ones obtained in Eq. (15)

24 $$ C_{1} = F ^{*} \left[ {\begin{array}{*{20}c} {e^{{ - i\pi \lambda z\mu_{j}^{2} }} } & \cdots & 0 & 0 \\ \vdots & \ddots & 0 & \vdots \\ 0 & \cdots & {e^{{ - i\pi \lambda z\mu_{j}^{2} }} } & 0 \\ 0 & 0 & 0 & {e^{{ - i\pi \lambda z\mu_{j}^{2} }} } \\ \end{array} } \right]\left[ {\begin{array}{*{20}c} 1 \\ 1 \\ 1 \\ 1 \\ 1 \\ \end{array} } \right]F ^{*} \left[ {\begin{array}{*{20}c} \vdots \\ \vdots \\ {e^{{ - i\pi \lambda z\mu_{j}^{2} }} } \\ \vdots \\ \vdots \\ \end{array} } \right]\left[ {\begin{array}{*{20}c} \vdots \\ \vdots \\ {e^{{i\frac{{\pi x_{j}^{2} }}{\lambda z}}} } \\ \vdots \\ \vdots \\ \end{array} } \right] $$

Using the same computation, one obtains the following:

25 $$ T^{ \oplus } *\left[ {\begin{array}{*{20}c} 0 \\ 0 \\ 1 \\ 0 \\ 0 \\ \end{array} } \right] = \left[ {\begin{array}{*{20}c} \vdots \\ \vdots \\ {e^{{ - i\frac{{\pi x_{j}^{2} }}{\lambda z}}} } \\ \vdots \\ \vdots \\ \end{array} } \right] $$

Similarly, we can find the value of:

26 $$ T^{ - 1} *\left[ {\begin{array}{*{20}c} 0 \\ 0 \\ 1 \\ 0 \\ 0 \\ \end{array} } \right] = \left[ {\begin{array}{*{20}c} \vdots \\ \vdots \\ {e^{{i\frac{{\pi x_{j}^{2} }}{\lambda z}}} } \\ \vdots \\ \vdots \\ \end{array} } \right] $$

Thus, after putting the value of Eq. (25) and (26) in Eq. (19) one can obtain the condition for convergence as a function of the free space propagation distance:

27 $$ E_{{in_{j} }} \left( {x_{j} } \right) = K{*}e^{{ i\frac{{2\pi x_{j}^{2} }}{\lambda z}}} *E_{{out_{j} }}^{ \oplus } \left( {x_{j} } \right) $$

where K is a constant.

### Lateral focal location tuning

If we tune the lateral location this means that Z = 0, i.e., we have only the scattering medium and no free space propagation. Also, since the focus will now be not exactly in the center the assumption of Eq. 8 is no longer valid. However, we can still use our technique to find the focus at the center, obtain the correction wavefront that produced this focus. Later for shifting the focus, we can multiply this wavefront with a linear phase (Fig. 11b) or we can shift the optimized pattern on the SLM which in turn will shift the focal spot because of the memory effect of speckles.

### Optimization protocol

**Figure 12:**
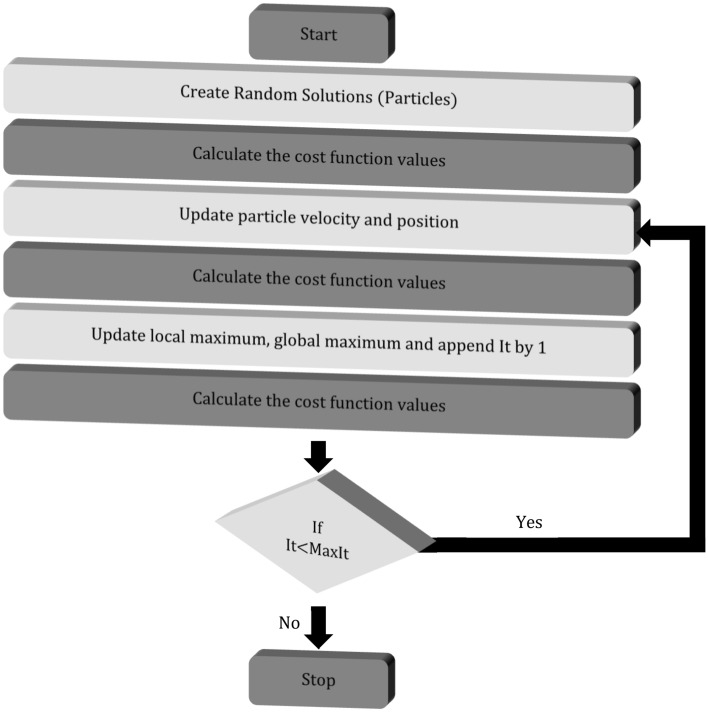
Flowchart describing the steps involved in Particle Swarm Optimization (PSO).

We used Particle Swarm Optimization Scheme (PSO) to perform the iterations. It is a stochastic optimization technique that mimics the social behavior of birds flocking together or fish schooling. It was introduced by Kennedy, Eberhart, and Shi^45^. PSO finds an optimum solution for a problem by iteratively improving the solution from a set of candidate solutions being random guess solutions to a problem. These random solutions are called *particles.* Each particle is assigned a *‘position’* and a *‘velocity’* vector. The search space is known as *‘swarm’*. Hence, the name PSO. The flowchart describing the steps involved in PSO is shown in Fig. 12. We optimize the Ein (which are random phase masks applied on SLM) using PSO to increase the cost function which in turn will focus light after the first pass through the medium.

### Conclusions

We have presented a novel technique to focus light through a scattering medium non-invasively. Uniqueness comes from the fact that we neither need to know the scattering properties a priori nor need to use any feedback mechanism from behind the medium. In the current proof of concept experiment we demonstrated that indeed, it is possible to tune and shift the focus axially and laterally by numerical optimization of the incident wavefront. This technique has a strong potential to make its impact in various fields such as biomedical imaging and diagnostics. The concept of reciprocity-induced symmetry and its implications has not been extensively explored. Hence, we hope that our technique in its current form can be further enhanced to implement it for various practical imaging applications.
