# Author Correction: Optical reciprocity induced wavefront shaping for axial and lateral shifting of focus through a scattering medium

**DOI:** 10.1038/s41598-022-12515-8

**Published:** 2022-05-17

**Authors:** Abhijit Sanjeev, Vismay Trivedi, Zeev Zalevsky

**Affiliations:** Faculty of Engineering and the Institute for Nanotechnology and Advanced Materials, Bar-Ilan University, 5290002 Ramat-Gan, Israel

Correction to: *Scientific Reports* https://doi.org/10.1038/s41598-022-10378-7, published online 16 April 2022

The original version of this Article contained errors in the in-text citations.

In the Introduction:

“However, this technique suffers from the fact that it requires a lot of image processing tools, and post processing^8^”

now reads:

“However, this technique suffers from the fact that it requires a lot of image processing tools, and post processing”

“Another interesting approach is to exploit the memory effect of speckles. Memory effect states that there exists a correlation between a speckle pattern even when the laser illumination is tilted within a given angular range. Freund^12,13^ used the scattering media as a lens based on speckle intensity correlation. This idea was extended by Bertolotti et al.^14^ to image a fluorescent object behind the media.”

now reads:

“Another interesting approach is to exploit the memory effect of speckles. Memory effect states that there exists a correlation between a speckle pattern even when the laser illumination is tilted within a given angular range. Freund^12–14^ used the scattering media as a lens based on speckle intensity correlation. This idea was extended by Bertolotti et al.^13^ to image a fluorescent object behind the media.”

“Despite the advantage of utilizing the scattered light without any wavefront correction, this approach suffers from the limited memory effect range and axial decorrelation of the speckle effect^18^”.

now reads:

“Despite the advantage of utilizing the scattered light without any wavefront correction, this approach suffers from the limited memory effect range and axial decorrelation of the speckle effect^16^”.

“There were several works done by using SLM as a phase compensator to image through a scattering medium^17,24–33^”

now reads:

“There were several works done by using SLM as a phase compensator to image through a scattering medium^18,24–33^”

In the Results, under the subheading “Simulation results”,

“Simulations were performed in MATLAB. We followed the modeling as proposed and implemented by Zhu *et al.*^8^.”

now reads:

“Simulations were performed in MATLAB. We followed the modeling as proposed and implemented by Zhu *et al.*^44^.”

In the Methods, under the subheading ‘Principle’,

“Recently Szu *et al.*^8^ proved this point experimentally in a disordered medium like a multimode fiber.”

now reads:

“Recently Lee *et al.*^8^ proved this point experimentally in a disordered medium like a multimode fiber.”

In the Methods, under the subheading “Optimization protocol”,

“It was introduced by Kennedy, Eberhart, and Shi^44^.”

now reads:

“It was introduced by Kennedy, Eberhart, and Shi^45^.”

In the References, Reference 16 and Reference 17 were interchanged.

The correct References are listed below:

16. Katz, O., Small, E. & Silberberg, Y. Looking around corners and through thin turbid layers in real time with scattered incoherent light. *Nat. Photon.* **6**, 549–553 (2012).

17. Singh, A. K., Pedrini, G., Takeda, M. & Osten, W. Scatter-plate microscope for lensless microscopy with diffraction-limited resolution. *Sci. Rep.* **7**, 10687 (2017).

In addition, the original version of this Article contained an error in the Figure legend of Figure 2. The descriptions of Panel (a) and Panel (b) were inadvertently switched.

The legend of Figure 2:

“Simulation Results showing (**a**) Scattered Speckle pattern on the last plane of the diffuser when it is illuminated with a plane wave, (**b**)The focus obtained after optimization when the diffuser is illuminated with the correct phase mask that cancels the diffuser to obtain a strong focus non-invasively. (**c**) Intensity profile along an axis for both (**a**) and (**b**) marked in red and blue respectively.”

now reads:

“Simulation Results showing (**a**) The focus obtained after optimization when the diffuser is illuminated with the correct phase mask that cancels the diffuser to obtain a strong focus non-invasively. (**b**) Scattered Speckle pattern on the last plane of the diffuser when it is illuminated with a plane wave, (**c**) Intensity profile along an axis for both (**a**) and (**b**) marked in blue and red respectively.”

The original Article has been corrected.

