# Publisher Correction: Optical reciprocity induced wavefront shaping for axial and lateral shifting of focus through a scattering medium

**DOI:** 10.1038/s41598-023-32998-3

**Published:** 2023-05-09

**Authors:** Abhijit Sanjeev, Vismay Trivedi, Zeev Zalevsky

**Affiliations:** Faculty of Engineering and the Institute for Nanotechnology and Advanced Materials, Bar-Ilan University, 5290002 Ramat-Gan, Israel

Correction to: *Scientific Reports* https://doi.org/10.1038/s41598-022-10378-7, published online 16 April 2022

The original version of this Article contained an error in Eq. 1, where the equation contained wrong placements of bracket, a misplaced matrix, and devoid of the power M.

**Figure:**
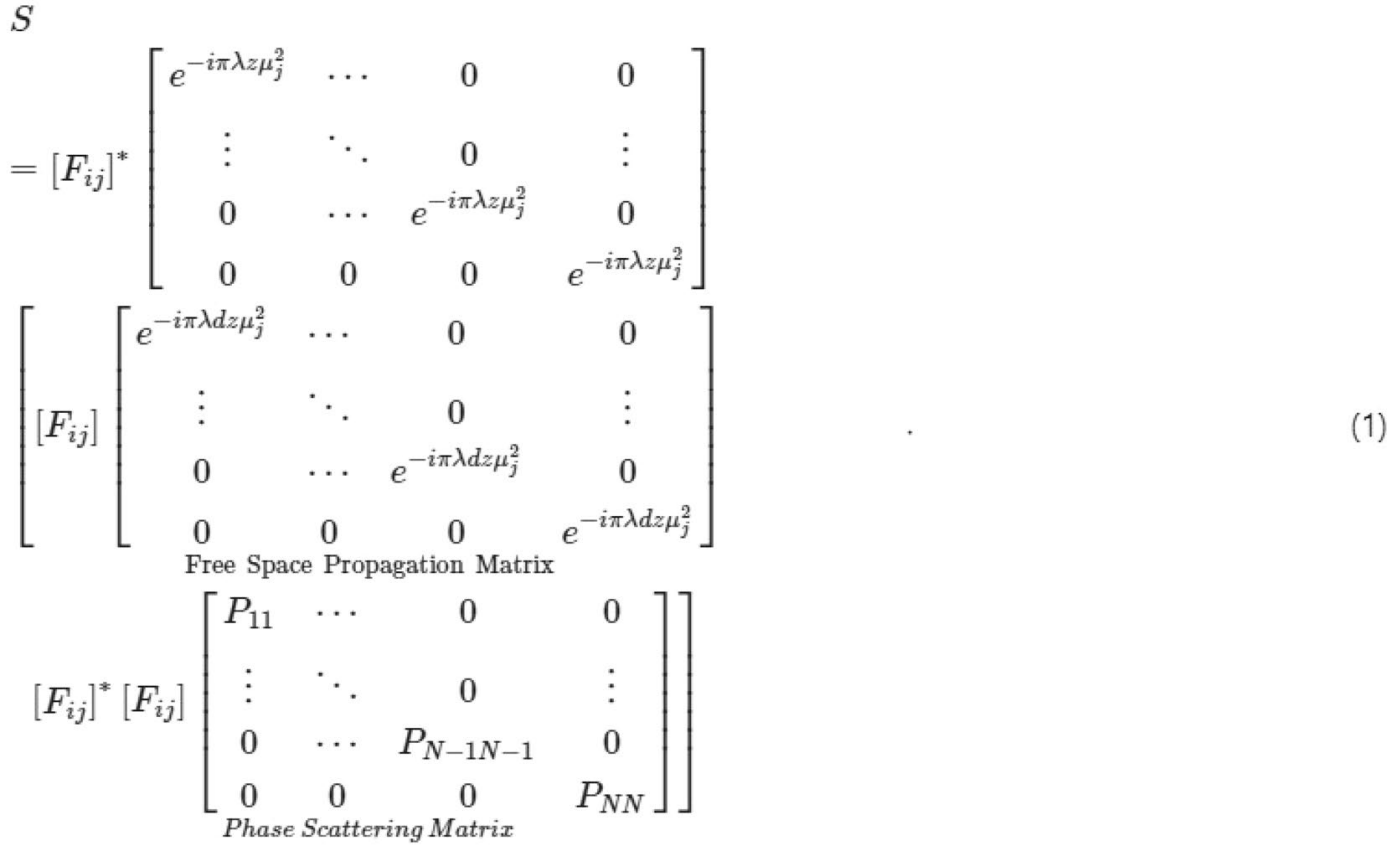

now reads:
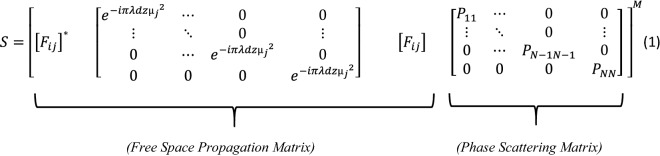


The original Article has been corrected.

